# Supplementary material for: Chromothripsis during telomere crisis is independent of NHEJ, and consistent with a replicative origin
Source: Genome Res. 2019 May;29(5):737–49. doi: 10.1101/gr.240705.118 (PMC6499312; doi:10.1101/gr.240705.118)
Supplement: Supplemental Material [file supp_gr.240705.118_Supplemental_file_1.zip › contigs/annotated_contigs/DB70/contig.2.DB70_length_485_mean_cov_22.1072164948.docx]

**DB70_length_485_mean_cov_22.1072164948**

```
CTCCCGGTTTCAAGCGATTCTCCTGCATCGGCCTCCTGAGTAGCCGGGATTGCGGGCATGCGCTGCCACGTCTGGCTGATTTCGTATTT
 >chrUn_gl000220:124335-124737 + E=1e-230 p=0e+00
TTAGTGGAGACGGGGCTTCTCCATGTCGATCGGGCTGGTTTCGAACTCCCGACCTCAGGTGATCCGCCCTCCCCGGCCTCCGGAAGTGC

TGGGATGACAGGCGTGAGCCACCGCGCCCGGCCTTCATTTTTAAATGTTTTCCCACAGACGGGGTCTCATCATTTCTTTGCAACCCTCC

TGCCCGGCGTCTCAAAGTGCTGGCGTGACGGGCGTGAGCCACTGCGCCTGGACTCCGGGGAATGACTCACGACCACCATCGCTCTACTG

ATCCTTTCTTTCTTTCTTTCTTTCTTTCTTTCTTTCTTTCTTTCTT|T|CTTTCTTTCTTTCTTTCTTTCTTTCTTGATGAAT|TATCT
                                                >chr19:46339169-46339203 - E=7e-06
TATGTTTTTTTTTTTTTCTTATTTTCTGTCGGCGTCTTGCTCT
```
